# Microtubule-associated protein light chain 3 is involved in melanogenesis via regulation of MITF expression in melanocytes

**DOI:** 10.1038/srep19914

**Published:** 2016-01-27

**Authors:** Woo Jin Yun, Eun-Young Kim, Ji-Eun Park, Soo Youn Jo, Seung Hyun Bang, Eun-Ju Chang, Sung Eun Chang

**Affiliations:** 1 Department of Dermatology, University of Ulsan College of Medicine, Asan Medical Center, Seoul, Korea; 2 Department of Biomedical Sciences, Asan Medical Center, University of Ulsan College of Medicine, Seoul, Korea

## Abstract

Although autophagy plays a role in melanogenesis by regulating melanosome degradation and biogenesis in melanocytes, a detailed understanding of the regulatory functions of autophagy factors is lacking. Here, we report a mechanistic link between microtubule-associated protein light chain 3 (LC3) activation and melanogenesis. We observed high expression of LC3 in melanosome-associated pigment-rich melanocytic nevi of sun-exposed skin, as indicated by patterns of melanosomal protein MART1 expression. Rapamycin-induced autophagy significantly increased the melanin index, tyrosinase activity and expression of several proteins linked to melanosome biogenesis, including microphthalmia transcription factor (MITF), pre-melanosome protein and tyrosinase, in Melan-a melanocytes. siRNA-mediated knockdown of *LC3*, but not *beclin-1* or *ATG5*, decreased melanin content and tyrosinase activity. LC3 knockdown also markedly inhibited MITF expression and subsequent rapamycin-induced melanosome formation. More importantly, LC3 knockdown suppressed α-MSH-mediated melanogenesis by attenuating cAMP response element-binding protein (CREB) phosphorylation and MITF expression in Melan-a cells via decreased extracellular signal-regulated kinase (ERK) activity. Overexpression of constitutively active ERK reversed the effect of LC3 knockdown on CREB phosphorylation and MITF expression. These findings demonstrate that LC3 contributes to melanogenesis by increasing ERK-dependent MITF expression, thereby providing a mechanistic insight into the signaling network that links autophagy to melanogenesis.

Melanogenesis is a key process for melanin production, which determines color of the skin, hair and eyes, and protects the skin from the harmful effects of sunlight, toxic drugs and chemicals^1^,^2^,^3^. Melanogenesis is regulated by multiple transcriptional, enzymatic and spatial control mechanisms; for example, microphthalmia-associated transcription factor (MITF) is critical for melanogenic enzyme transcription^4^,^5^. Several studies have also reported the importance of MITF in melanocyte development and survival^6^, where it enhances the expression of tyrosinases, such as tyrosine-related protein 1 (TRP-1) and TPR-2^7^. The TRP-1 and TRP-2 activation pathway is mainly involved in transformation of tyrosine to melanin pigments^8^. In addition, MITF directly regulates expression of the melanogenic gene *PMEL17*^9^, whose product contributes to melanogenesis by inducing formation of functional fibrils^10^.

Autophagy is essential for tissue homeostasis, adaption to starvation, and removal of dysfunctional organelles or pathogens^11^. Autophagy regulators may have prominent roles in the initial stages of melanosome formation, a lysosome-related organelle in which melanin is synthesized^12^,^13^. Beclin-1^14^, autophagic modulator WIPI1 (mammalian homolog of ATG18)^15^ and LC3 (microtubule-associated protein light chain 3) are potent regulators of melanogensis in melanocytic lesions^16^. Co-localization of LC3 in melanosomes with amyloid protein PMEL17^17^,^18^ highlights the importance of the autophagy pathway in regulation of melanosome biogenesis. However, little is known about the underlying mechanisms of melanogenesis. Based on these previous findings, we hypothesized that a mechanistic link might exist between certain autophagic factors and melanogenic gene expression during melanogenesis.

## Results

### Autophagy activation correlates with melanogenesis

**Figure 1:**
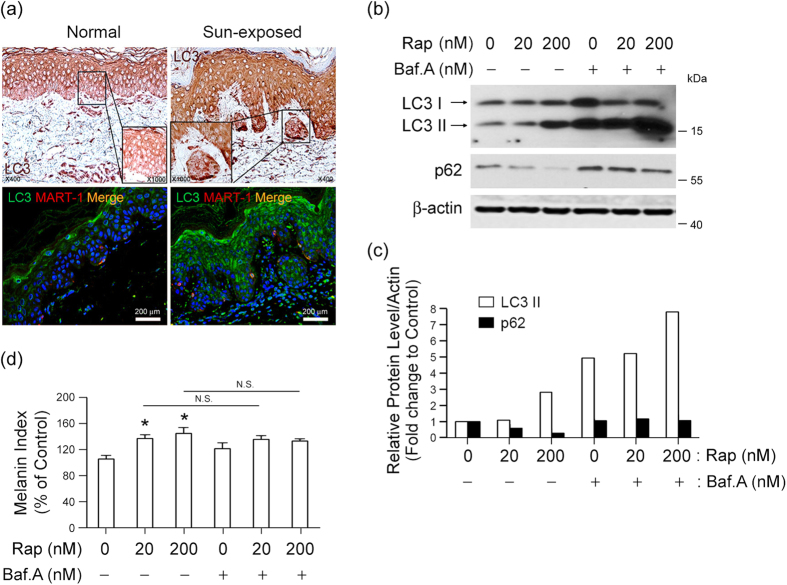
Autophagy activation correlates with melanogenesis in melanocytic nevi. **(a)** LC3 expression coincides with melanogenesis in sun-exposed skin of Korean patients. Immunohistochemical (top) or immunofluorescence staining (bottom) with anti-LC3 antibody (green) and anti-MART1 (melan A; red) antibody was performed to determine the extent to which LC3 expression is associated with melanin pigment in sun-exposed skin. The inset box represents an area shown at higher magnification (1000×). Magnification, 400×; Scale bar, 200 μm. **(b–d)** The relationship between autophagy flow and melanin synthesis in Melan-a melanocytes in the presence or absence of autophagy activator rapamycin (Rap) at indicated concentration for 24 h was determined by detecting conversion of LC3-I to LC3-II and p62 degradation using Western blot analysis. A subset of cells was also exposed to the lysosomal inhibitor bafilomycin A1 (Baf.A). Protein expression was determined via quantitative densitometry of experimental proteins compared to β-actin expression **(b,c)**, and melanogenesis was estimated by measuring melanin content **(d)** compared to control cells. *P < 0.05 versus untreated controls. N.S., not significant.

Autophagy is activated to maintain cellular homeostasis under various stress conditions^19^. We found that expression of LC3, a representative autophagy protein, was elevated, and the protein was co-localized with MART1 (melan A) in melanocytic nevi, as detected by immunohistochemical staining (Fig. 1a; upper panels) and immunofluorescence analysis (Fig. 1a; lower panels) in the nests of pigmented nevi of sun-exposed skin (Fig. 1a; right panel), but not in the basal or supra-basal layer of normal skin or epidermis (Fig. 1a; left panel). This observation indicates that autophagy activation takes place in melanogenic regions. We next investigated if autophagy activation is linked to melanogenesis by determining the melanin index of murine Melan-a melanocytes. We assessed autophagy activation by tracking the conversion of LC3-I to LC3-II^18^, which can be precisely determined by preventing autophagosome–lysosome fusion and subsequent autophagosomal degradation using a lysosomal inhibitor, such as bafilomycin A1. Thus, we compared LC3-II levels in the presence or absence of bafilomycin A1 and observed an accumulation of LC3-II in its presence (Fig. 1b,c). The autophagy activator rapamycin increased LC3-II expression in association with decreased expression of p62, a known substrate for autophagic degradation. Rapamycin-induced autophagy also enhanced melanin synthesis in Melan-a melanocytes with no significant differences in melanin indices between bafilomycin A1-treated or non-treated cells (Fig. 1d), indicating that melanogenesis in these cells is associated with LC3-II conversion during autophagy.

**Figure 2:**
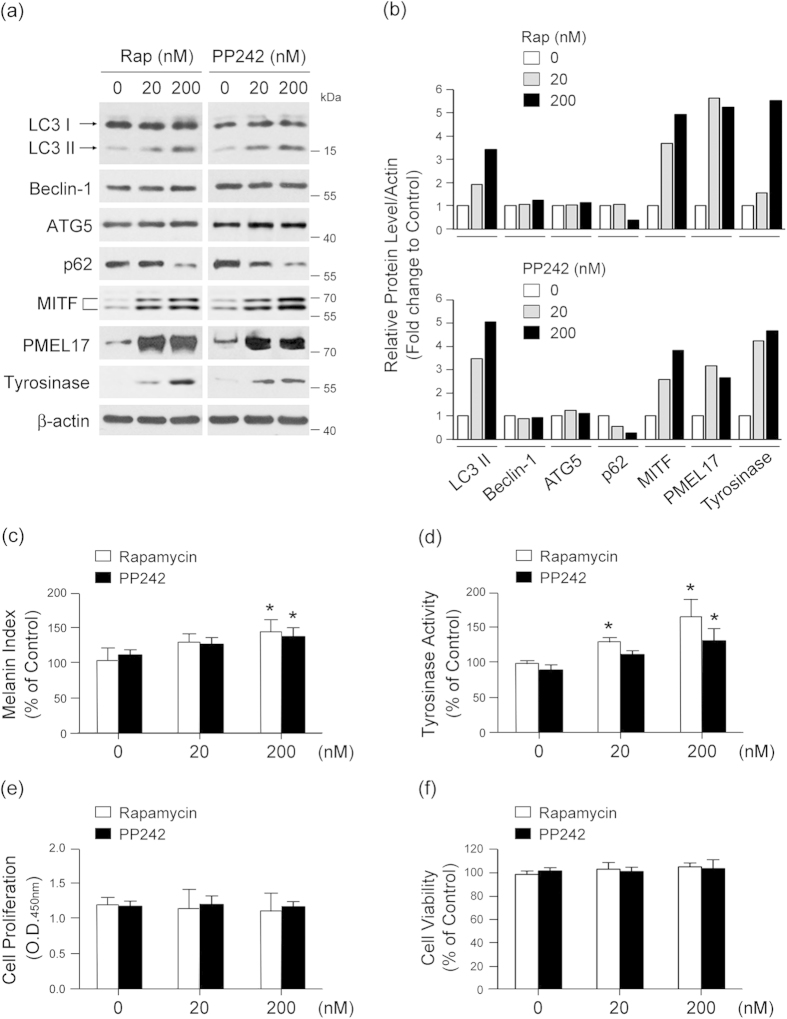
Autophagy induction promotes melanogenesis. **(a,b)** Melan-a melanocytes were treated with indicated concentration of rapamycin (Rap) or PP242 for 24 h and analyzed by Western blotting with antibodies against LC3, beclin-1, ATG5, p62, MITF, PMEL17, tyrosinase, and β-actin. Quantitative densitometry of protein expression compared to β-actin is shown. To determine the effect of autophagy activation on melanogenesis, cells were assayed for melanin content **(c)** and tyrosinase activity **(d)**. The effect of Rap and PP242 on cell proliferation **(e)** and viability **(f)** was determined at 24 h post-treatment. All results shown are the mean of three independent experiments ± SD. *P < 0.05 versus untreated controls.

To further explore the relationship between autophagy activation and melanin synthesis, we compared LC3-1/II conversion and expression levels of p62 and melanogenesis-associated proteins MITF, PEML17, and tyrosinase^20^,^21^ after exposure to rapamycin or PP242, an mTOR inhibitor^22^. Both compounds increased endogenous LC3-I/LC3-II conversion, decreased levels of p62, and increased expression of MITF, PMEL17, and tyrosinase (Fig. 2a,b). Rapamycin and PP242 also significantly increased melanin synthesis and tyrosinase activity in a dose-dependent manner (Fig. 2c,d) without affecting proliferation (Fig. 2e) or viability (Fig. 2f) of the melanocytes. These results support the notion that autophagy activation may induce melanogenesis in these cells.

### LC3 is required for autophagy-induced melanogenesis by regulating MITF expression

**Figure 3:**
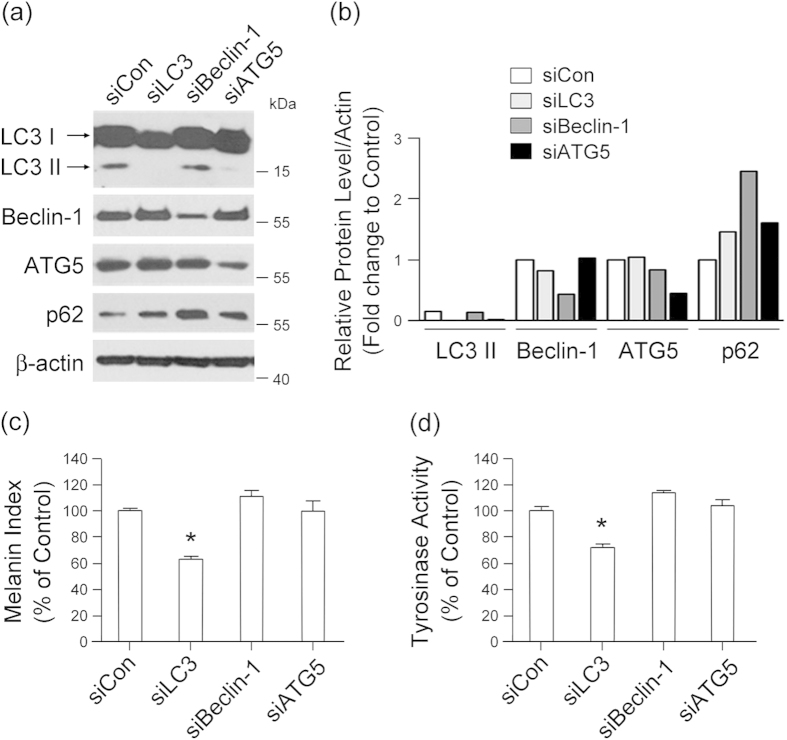
LC3 activation is required for melanogenesis. Inhibition of LC3 attenuates melanin synthesis and tyrosinase activity in melanocytes. Melan-a cells were transfected with specific siRNAs for LC3, beclin-1, ATG5, or control siRNA for 24 h and were analyzed by Western blotting after autophagy induction **(a,b)**. Quantification of melanin **(c)** and tyrosinase activity **(d)** was performed for each transfected cell line. Results shown are the mean of three independent experiments ± SD. *P < 0.05 versus untreated controls.

**Figure 4:**
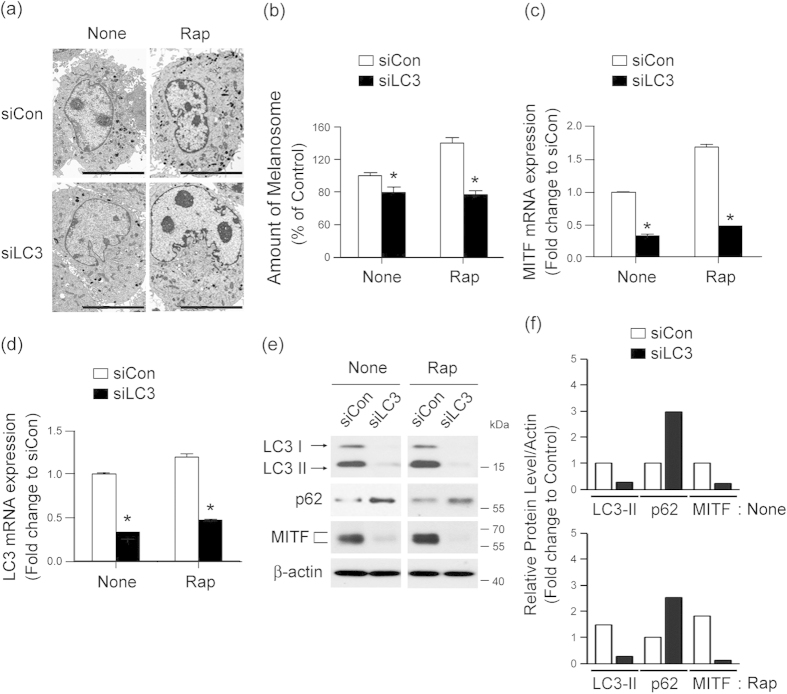
LC3 activation involves MITF expression during autophagy-induced melanogenesis. **(a–f)** Knockdown of LC3 decreases autophagy-induced melanogenesis. Melan-a cells were transfected with control siRNA (siCon) or LC3-specific siRNA (siLC3) and treated with (Rap) or without (None). Loss of Rap-induced melanosome formation following transfection with LC3 siRNA compared with control cells was observed by electron microscopy **(a)** and quantified (**b)**. Scale bar, 5 μm. Data represent ± standard error of the mean (SEM) (*P < 0.05). After autophagy activation, quantitative real-time PCR of *MITF* **(c)** and *LC3* **(d)**, Western blot analysis of LC3, p62, MITF and β-actin **(e)** and quantitative densitometry of proteins compared to β-actin **(f)** were performed. Results shown are the mean of three independent experiments ± SD. *P < 0.05 versus the siRNA control group.

LC3, beclin-1 and autophagy-related protein 5 (ATG5) are essential proteins for autophagy^23^. To define which autophagy-related genes may be involved in autophagy-induced melanogenesis, we tested the effect of siRNA-silenced *LC3*, *beclin-1*, and *ATG5* on melanogenesis in Melan-a melanocytes. Knockdown of *LC3* by siRNA transfection (Fig. 3a,b) resulted in marked attenuation of melanin synthesis (Fig. 3c) and tyrosinase activity (Fig. 3d). In contrast, down-regulation of *beclin-1* or *ATG5* did not significantly suppress the melanin index of Melan-a cells (Fig. 3a–d), emphasizing the association of LC3 with melanogenesis. To verify the involvement of LC3 in autophagy-induced melanogenesis, we directly investigated cellular melanosomes by electron microscopy and determined levels of melanin synthesis by melanin indexing. Treatment with rapamycin induced melanosome formation and accumulation of melanin in Melan-a cells transfected with control siRNA, while the number of melanosomes was significantly reduced after LC3 knockdown (Fig. 4a,b). Furthermore, expression of MITF significantly decreased in LC3-siRNA-transfected Melan-a cells at the transcriptional (Fig. 4c,d) and translational (Fig. 4e,f) level with or without rapamycin treatment, suggesting the involvement of LC3 in regulating MITF expression.

### LC3 potentiates melanogenic signal induced by α-MSH by modulating ERK-CREB pathway

**Figure 5:**
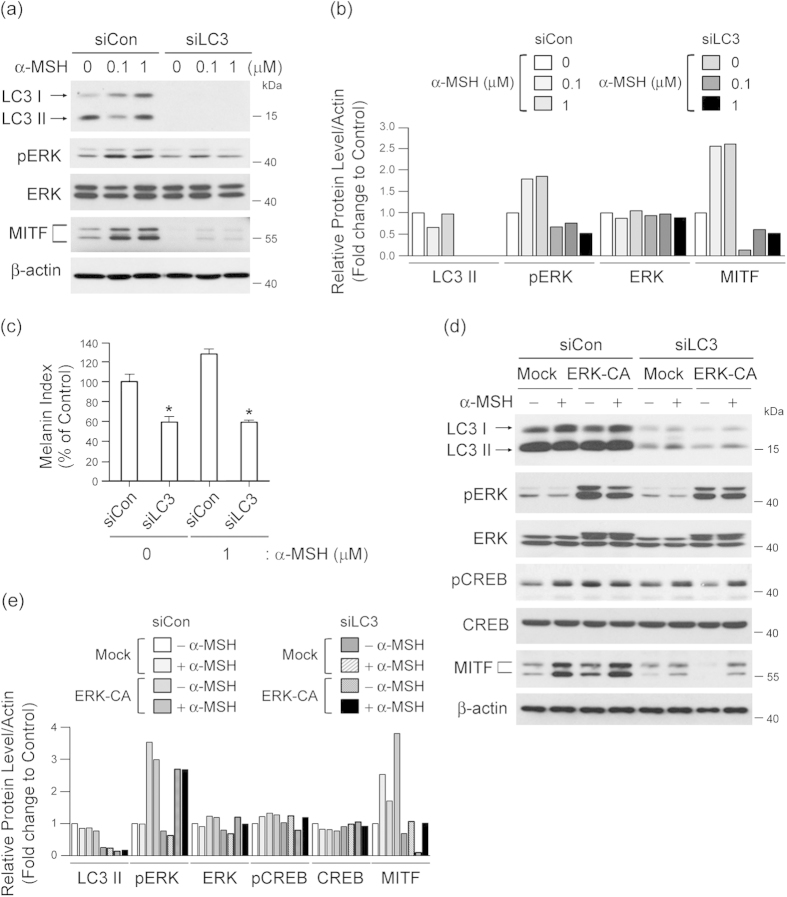
LC3 modulates α-MSH-induced ERK and CREB activation, resulting in MITF expression. Knockdown of LC3 decreases α-MSH-induced melanogenesis. Melan-a cells transfected with control siRNA (siCon) or LC3 siRNA (siLC3) were treated with or without α-MSH (0.1 or 1 μM), and expression of LC3-I/II, pERK, ERK, MITF, and β-actin via Western blot and densitometry was measured **(a,b)**. The melanin index of treated versus control cells is also shown **(c).** Results shown are the mean of three independent experiments ± SD. *P < 0.05 versus siRNA-treated control group. **(d,e)** Inhibition of LC3 suppresses MITF expression via ERK-dependent CREB activation. Melan-a cells co-transfected with siCon or siLC3 and pcDNA5 vector DNA (Mock) or pcDNA5-ERK-CA (constitutive ERK active form; ERK-CA) were treated with or without α-MSH for 24 h. The cell lysates were collected and analyzed by Western blot analysis to quantify expression of MITF, LC3,pERK, ERK, pCREB, CREB, and β-actin **(d)**, as well as by quantitative densitometry of protein levels compared to β-actin **(e)**.

α-melanocyte-stimulating hormone (α-MSH), through activation of its receptor, melanocortin-1 receptor (MCR-1), is a representative stimulator of melanogenesis by inducing MITF expression^2^,^24^. Thus, we surmised that an LC3 may modulate α-MSH-mediated MITF expression and subsequent melanogenesis. To explore this possibility, we next investigated the regulatory role of LC3 in α-MSH-mediated signaling, such as ERK and cAMP response element-binding protein (CREB), related to MITF expression^25^,^26^. We found that α-MSH treatment induced phosphorylation of ERK and increased MITF expression in Melan-a cells transfected with control siRNA (Fig. 5a,b) that coincided with increased melanin synthesis (Fig. 5c). However, these effects were eliminated by LC3 knockdown (Fig. 5a,b). Moreover, overexpression of constitutively active ERK restored CREB phosphorylation and MITF expression evoked by LC3 knockdown (Fig. 5d,e), indicating that LC3-corrleted MITF expression acts through an ERK-CREB pathway that interacts with α-MSH-induced melanogenic signaling.

**Figure 6:**
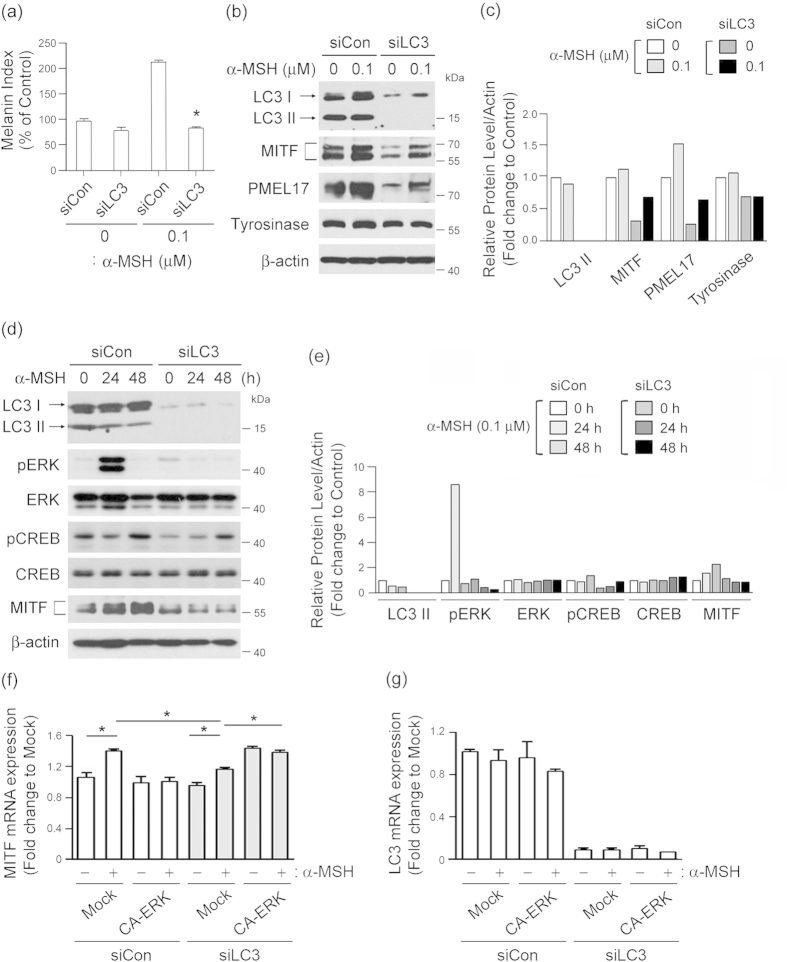
LC3 activation potentiates the melanogenic signals of α-MSH-sensitive melanoma cells. **(a)** Inhibition of LC3 suppresses α-MSH-induced melanogenesis. B16F10 melanoma cells transfected with control siRNA (siCon) or LC3 siRNA (siLC3) were treated with or without 0.1 μΜ α-MSH, after which their melanin content was measured. Results shown are the mean of three independent experiments ± SD. *P < 0.05 versus siRNA-treated control group. **(b,c)** LC3 knockdown inhibits α-MSH-induced signaling. B16F10 cells transfected with or without LC3 siRNA were stimulated with indicated concentrations α-MSH for 24 h, and expression levels of LC3-I/II, MITF, PMEL17 and tyrosinase were examined by Western blotting **(b)**. Quantitative densitometry of protein levels compared to β-actin is shown **(c)**. **(d,e)** Inhibition of LC3 suppresses α-MSH-induced ERK and CREB activation. B16F10 cells transfected with siCon or siLC3 for 24 h were treated with or without α-MSH for 24 h or 48 h, followed by Western blot analysis of LC3, pERK, ERK, pCREB, CREB, MITF and β-actin expression **(d)** and quantitative densitometry of protein levels compared to β-actin **(e)**. **(f,g)** B16F10 melanoma cells were co-transfected with siCon or siLC3 and pcDNA5 vector DNA (Mock) or pcDNA5-ERK-CA (constitutive ERK active form; ERK-CA) and treated with or without α-MSH for 24 h. The cell lysates were analyzed by quantitative real-time PCR for *MITF* and *LC3* expression. Results shown are the mean of three independent experiments ± SD. *P < 0.05 versus siRNA-treated control group.

**Figure 7:**
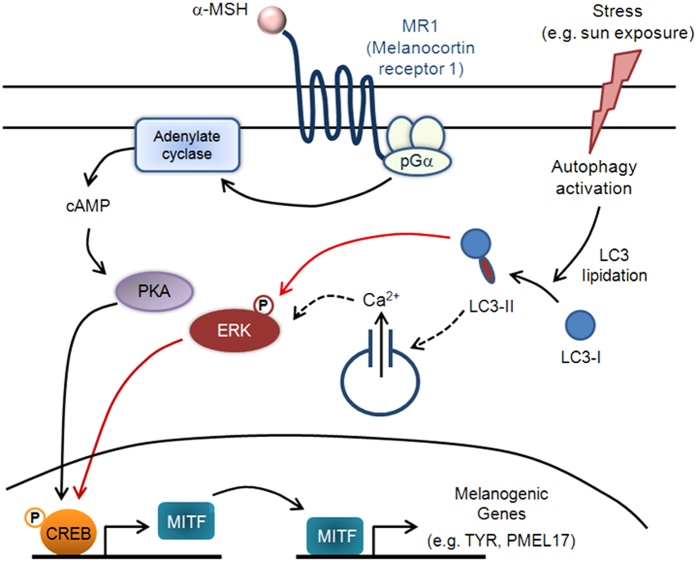
Proposed model of LC3’s role in melanogenesis during stress response. This schematic describes our proposed model of the signaling pathways involved in melanogenesis and their potential link to autophagy activation. Black arrows depict steps that have been experimentally verified from previous reports, whereas dashed arrows indicate unconfirmed steps. Red arrows represent experimental results from our current study: (**i**) Autophagy activation increases LC3-II levels, which triggers ERK and CREB phosphorylation and (**ii**) ERK-CREB activation leads to increased MITF expression and subsequent melanogenesis. The results reported here suggest that LC3 is a mediator that potentiates melanogenic signaling via α-MSH and MITF under certain stress conditions (e.g. autophagy activation) in melanocytes.

We also confirmed the role of LC3 in MITF induction upon melanogenic stimuli using B16F10 melanoma cells that are sensitive to α-MSH challange^27^. As shown in Fig. 6a, after α-MSH stimulation, melanin content was markedly higher in control siRNA-treated cells than in LC3 siRNA-treated cells. LC3 knockdown drastically attenuated expression of MITF and PMEL17 initially stimulated by α-MSH (Fig. 6b,c), indicating that LC3 knockdown diminishes the α-MSH-induced melanogenesis. In addition, cell treatment with α-MSH induced phosphorylation of ERK and CREB in control siRNA-treated cells, while α-MSH-induced ERK and CREB activation did not occur in LC3 siRNA-treated cells (Fig. 6d,e), emphasizing that ERK-CREB activation is a downstream signaling component in the pathway involved in LC3-mediated MITF expression^28^,^29^. Finally, we confirmed that ectopic expression of constitutively active ERK elevated MITF expression (Fig. 6f), even after LC3 knockdown in B16F10 cells (Fig. 6g). Taken together, these results suggest that LC3 can potentiate an α-MSH-mediated signaling pathway by enhancing ERK-CREB activation, which in turn leads to increased MITF expression and initiation of melanogenesis (Fig. 7).

## Discussion

Based on a previous report showing that autophagy activation occurs during melanosome biogenesis^13^, we hypothesized that LC3 may be an intracellular mediator of melanogenesis. The present study proposes that autophagy activation enhances melanin synthesis and correlates with levels of LC3-II in melanocytes. Interestingly, we observed that the cells’ melanin indices did not significantly differ in melanocytes when treated with the autophagy inhibitor bafilomycin A1. Moreover, despite activation of the autophagic degradation pathway as indicated by p62 degradation, the cells’ melanin indices were maintained. Therefore, the high level of melanin synthesis we observed in the cells does not appear to be associated with blockage of autophagic degradation in melanocytes but may be correlated with LC3-II expression.

In support of this finding, we also determined that autophagy-associated melanogenesis after rapamycin treatment was mediated by LC3, but not by beclin-1 or ATG5, further indicating that LC3 is a specific intracellular mediator of autophagy-induced melanogenesis independent of other autophagy-related proteins. During autophagic degradation, ATG5 and beclin-1 mainly participate in autophagosome formation, a process involving degradation of protein aggregates, and in the recycling of macromolecules through fusion with lysosomes^29^,^30^. In contrast, LC3 mediates diverse cellular functions in various cells as a signaling molecule distinct from other autophagic factors by associating with microtubules^31^ and localizing to nuclei^32^. Our current study also showed that LC3 depletion suppresses MITF expression in accordance with decreased melanin content and tyrosinase activity, even in rapamycin-treated cells. Depletion of beclin-1 inhibits accumulation of pigments, LC3-II and melanosomes *in vitro* and *in vivo* without affecting levels of MITF and tyrosinase mRNA^20^. The above results indicate that not all autophagy regulators control MITF and tyrosinase transcription and that mechanisms guiding autophagic regulator proteins during melanogenic signaling may vary with respect to cell type or environmental stimuli^20^. Because our current study focuses on the role of LC3 as a signaling modulator in melanin synthesis, future studies should explore the underlying molecular mechanism(s) by which LC3 contributes to melanosome biogenesis and/or degradation and to what extent intrinsic LC3-induced melanogenic capacity promotes melanin secretion.

MITF is linked to melanogenic potential via an MITF-mediated increase of tyrosinase activity^33^. Depletion of the serine/threonine kinase mTOR or pharmacologic inhibition of complex TORC1 leads to increased MITF transcription, whereas depletion of autophagy protein WIPI1 increases TORC1 activity, which leads to repression of TORC2, activation of GSK3β, increased β-catenin degradation, and decreased MITF transcription^20^. The present study showed that MITF is a critical mediator of LC3-II-dependent melanogenesis during rapamycin-induced autophagy. We also found that LC3-II enhanced α-MSH-dependent melanogenesis by increasing MITF expression via its ability to activate ERK. Furthermore, knockdown of LC3 inhibited MITF expression, even in the presence of α-MSH stimulation. LC3-II likely promotes ERK phosphorylation^24^, thus initiating a pathway that transduces an upstream signal for α-MSH-induced MITF expression during melanogenesis. We suggest that these signals potentiate α-MSH-mediated CREB phosphorylation and MITF expression, which contributes to melanogenesis. One possible mechanistic explanation of autophagy-induced melanogenesis is that mobilization Ca^2+^ via α-MSH activation leads to induction of ERK activity and autophagy^34^,^35^,^36^,^37^, supported by a previous study^39^ that showed rapamycin elicited autophagy and remodeled intracellular Ca^2+^ signaling machinery in HeLa cells. In addition, recent findings have suggested Ca^2+^ activates CREB, a well-characterized target of MITF, via the Rap1-ERK pathway during normal neuronal function^26^. Furthermore, α-MSH induces cAMP production via activation of adenylcyclase and phosphorylation of CREB, the latter of which directly stimulates MITF transcription and ultimately contributes to melanogenesis^38^,^39^. Therefore, multiple relationships between intracellular Ca^2+^ signaling and autophagy and with subsequent CREB/MITF activation via ERK activity may influence melanogenesis. Another explanation of our findings relates to the possible role of nuclear LC3 in response to melanogenic stimuli, as LC3 localizes to the nucleus and regulates autophagy^32^,^40^. Because we detected α-MSH-mediated LC3-II nuclear localization in B16F10 cells (data not shown), it is plausible that LC3-II localization influences transcriptional regulation of MITF; however, the relevance of its localization with respect to MITF expression in melanocytes requires further investigation.

In conclusion, the proposed model presented in Fig. 7 summarizes the role of LC3 in the promotion of melanogenesis. LC3-II-dependent ERK activation signals participate in and/or modulate CREB phosphorylation and MITF expression that links autophagic and α-MSH-induced melanogenic pathways under certain stress conditions, such as sun exposure. Activation of stress-mediated autophagy triggers lipidation of LC3 to form LC3-II in melanocytes, which induced phosphorylation of ERK, likely through an increased intracellular Ca^2+^ release^26^,^34^,^37^. Once in the presence of melanogenic stimuli, such as α-MSH, LC3-II-dependent ERK activation increases CREB phosphorylation of CREB and subsequent MITF expression, leading to induction of melanogenic gene expression (e.g. *TYR*, *PMEL17*). Thus, LC3-II, elevated during autophagy activation, appears to participate in and/or modulate a mechanism that links autophagic and melanogenic pathways under certain stress conditions.

## Methods

### Reagents and cell culture

Rapamycin, PP242, and bafilomycin A1 were purchased from Sigma-Aldrich (St. Louis, MO, USA). pcDNA5 (Mock) and pcDNA5-ERK-CA (constitutive ERK active form) were a kindly provided from Dr. Choi kyung-Cheol (University of Ulsan College of Medicine, Seoul, South Korea). Melan-a cells were mouse-derived, spontaneously immortalized melanocytes that synthesize large quantities of melanin. The cells were maintained in DMEM supplemented with 10% FBS, 100 nM TPA, 50 μg/mL streptomycin and 50 U/mL penicillin at 37 °C in 5% CO_2_. B16F10 murine melanoma cells were cultured in DMEM supplemented with 10% FBS, 50 μg/mL streptomycin and 50 U/mL penicillin at 37 °C in 5% CO_2_.

### Transient siRNA transfection

Melan-a or B16F10 cells were placed in 6-well plates, seeded at a quantity of 1 × 10^5^ cells, and transfected with siRNA against LC3, beclin-1, ATG5 or negative control siRNA using Lipofectamine™ RNAiMAX (Invitrogen, Merelbeke, Belgium) according to the manufacturer’s instructions. For overexpression of the constitutively active form of ERK, cells were co-transfected with control (siCon), LC3-specific siRNA (siLC3) and with 1 μg pcDNA5 vector DNA (Mock) or pcDNA5-ERK-CA (constitutive ERK active form; ERK-CA). After 24 h of transfection, the culture medium was replaced with fresh medium containing PBS, rapamycin (200 nM) or α-MSH (0.1 μM or 1 μM) and then incubated for an additional 24 h.

### Cell viability and growth assays

Cell viability was assessed using MTT (3-(4,5-Dimethylthiazol-2-yl)-2,5-diphenyltetrazolium bromide) assay kit (R&D Systems, Minneapolis, MN, USA) and measuring absorbance at 570 nm. Melan-a cells (5 × 10^4^) were cultured on 96-well plates and treated with 0, 20, or 200 nM rapamycin or PP242 for 24 h and counted using the MTT assay. Cell growth was evaluated with the CCK-8 colorimetric assay (Dojindo Molecular Technologies, Japan). Briefly, 10 μL cell CCK-8 solution was added to each well, and the plates were incubated for an additional 1–4 h at 37 °C. The absorbance at 450 nm of each aliquot was determined using a microplate reader. All analyses were performed in triplicate.

### Quantitative real time-PCR

RNA was extracted from cells using Trizol reagent (Ambion/Thermo-Fisher Scientific, Grand Island, NY, USA) according to the manufacturer’s instructions. For cDNA synthesis, 1 μg RNA was transcribed using the SuperScript First-Strand Synthesis Kit (Invitrogen, Carlsbad, CA, USA). Quantitative real-time PCR was performed using *Power* SYBR green PCR Master Mix (Applied Biosystems, Foster City, CA, USA) according to the manufacturer’s protocol on a LightCycler^®^ 480 II (Roche, West Sussex, UK). Primers used include the following: LC3 forward primer: 5′-CCCACCAAGATCCCAGTGAT-3′; LC3 reverse primer: 5′-CCAGGAACTTGGTCTTGTCCA-3′; MITF forward primer: 5′-ACTTTCCCTTATCCCATCCACC -3′; MITF reverse primer: 5′-TGAGATCCAGAGTTGTCGTACA-3′; GAPDH forward primer: 5′-TGGCCTTCCGTGTTCCTAC-3′; GAPDH reverse primer: 5′-GAGTTGCTGTTGAAGTTGCA-3′. GAPDH was used as a control to normalize amounts of cDNA among samples. Differences were calculated using the threshold cycle (Ct) and comparative Ct methods for relative quantification. Results were expressed as the relative expression of mRNA levels compared to controls.

### Melanin content and tyrosinase activity assays

The melanin content of cultured melanocytes was measured in accordance with the method described in a previous report^41^. Briefly, melanin from cell pellets was solubilized in boiled 1 M NaOH (80 °C) for 2 h, and the absorbance of the solution was measured at 405 nm. Tyrosinase activity was analyzed using the method described by Busca *et al.*^4^ with slight modifications. Cells were seeded in 6-well plates and incubated with rapamycin or α-MSH for 3-4 d. The cells were washed with ice-cold PBS, lysed with phosphate buffer (pH 6.8) containing 1% Triton X-100 and disrupted by freezing and thawing. After quantifying protein levels of the lysates and adjusting the protein concentrations with lysis buffer, 90 μL individual standardized lysates were placed in each well of a 96-well plate, and 10 μL 10 mM L-DOPA was then added. Control wells contained 90 μL lysis buffer and 10 μL 10 mM L-DOPA only. Following incubation at 37 °C for 20 min, the absorbance of each well was measured at 475 nm using an ELISA reader.

### Western blot analysis

For immunoblot analysis, whole-cell protein lysates were prepared and analyzed via Western blotting. Cells (2 × 10^5^ cells) were treated with indicated concentration of rapamycin or PP242 for 24 h in the absence or presence of 10 nM of bafilomycin A1 (Baf.A) and then were collected and lysed with buffer containing 40 mM Tris·HCl, pH 8.0, 120 mM NaCl, 0.1% Nonidet-P40, 100 mM phenylmethylsulfonyl fluoride, 1 mM sodium orthovanadate, 2 μg/mL leupeptin and 2 μg/mL aprotinin). Proteins were separated by SDS-polyacrylamide gel electrophoresis and transferred to a nitrocellulose membrane. The membrane was blocked with 5% nonfat dry milk in Tris-buffered saline and incubated with primary antibodies against: LC3 and β-actin (Sigma-Aldrich); ATG5 (Novus Biologicals, Littleton, CO, USA); p62 (Medical & Biological Laboratories, Nagoya, Japan); beclin-1, pERK, ERK, pCREB and CREB (Cell Signaling Technology, Beverly, MA, USA); and tyrosinase, PMEL17 and MITF (Santa Cruz Biotechnology, Santa Cruz, CA, USA) overnight at 4 °C. Blots were developed with a peroxidase-conjugated secondary antibody, and proteins were visualized by enhanced chemiluminescence with the Supersignal® West Femto Maximum Sensitivity Substrate Kit (Thermo-Fisher Scientific). Band densities were quantified using Image-J software.

### Immunohistochemistry

Patient biopsies were obtained with written informed consent and in accordance with the Helsinki declaration. The study was approved by the institutional review board of the Asan Medical Center (2014-0403). Five sets of biopsy tissues were fixed in 10% buffered neutral formalin and embedded in paraffin. The specimens were cut into 4-μm-thick sections, and serial sections were prepared for immunohistochemistry. For antigen retrieval, sections were autoclaved in antigen unmasking solution (Vector Laboratories, Burlingame, CA, USA) and stained for immunohistochemistry using the Vector Elite ABC Kit (Vector Laboratories) according to the manufacturer’s instructions. Sections were treated with diaminobenzidine (0.5 mg/mL) and hydrogen peroxide. Primary antibodies included anti-LC3 (1:100 dilution; Santa Cruz Biotechnology), anti-MART1 (1:300 dilution; Medical & Biological Laboratories, Nagoya, Japan) and mouse IgG1/kappa (1:50 dilution; eBioscience, Inc., San Diego, CA, USA). Slides were evaluated with confocal microscopy independently by two dermatologists with no knowledge of the patient’s identity or clinical outcome. Formalin-fixed, paraffin-embedded skin tissue sections were also used for immunofluorescent staining. The skin tissues were blocked with 1% BSA in PBS for 1 h and incubated with their respective primary antibodies for 2 h. After washing, the cells were stained with DAPI (Invitrogen) or dye-conjugated Alexar-Fluor 488 (for LC3; Santa Cruz Biotechnology) or Alexar-Fluor 649 (for MART-1; Abcam, Cambridge, UK) secondary antibodies. Sections were then mounted, and localization of LC3 and MART1 was detected using a confocal laser scanning microscope (LSM 710; Carl Zeiss, Tokyo, Japan).

### Electron microscopy

LC3 siRNA- or control siRNA-transfected melanocytes were treated with rapamycin for 24 h and prepared for electron microscopic analysis. Briefly, cultured cells were detached and fixed in a mixture of 4% paraformaldehyde and 2% glutaraldehyde. After post-fixation with 2% osmium tetroxide for 2 h, cells were dehydrated in ethanol and embedded in EPON resin. A 300-nm section of sample, stained with 2% uranyl acetate and lead citrate, was examined in a Tecnai 10 transmission electron microscope (Fei, The Netherlands). Structural changes associated with autophagy were observed as electron-dense matter enclosed in a double-membrane structure, a hallmark of the autophagosome.

### Statistical analyses

Numerical data were expressed as means ± standard deviation (SD) of independent determinations. A *P*-value < 0.05 was considered statistically significant. Statistically significant differences between groups were assessed using analysis of variance (ANOVA) and the Student’s *t* test.

## Additional Information

**How to cite this article**: Youn, W. J. *et al.* Microtubule-associated protein light chain 3 is involved in melanogenesis via regulation of MITF expression in melanocytes. *Sci. Rep.* **6**, 19914; doi: 10.1038/srep19914 (2016).
